# 2081: Phenotypic characterization of the CD4+ T-cell response during anti-CTLA4 therapy with ipilimumab in melanoma patients

**DOI:** 10.1017/cts.2017.199

**Published:** 2018-05-10

**Authors:** Farha Sherani, Duane Moogk, Anuj Bapodra, Karolina Malecek, Una Moran, Yesung Lee, Iman Osman, Michelle Krogsgaard

## Abstract

OBJECTIVES/SPECIFIC AIMS: To characterize the CD4+ T-cell response during CTLA-4 blockade immunotherapy with ipilimumab in patients with metastatic melanoma by correlating cytokine profiles with phenotypic changes in the intratumoral lymphocyte compartment of tumor biopsies obtained before and after treatment. METHODS/STUDY POPULATION: Peripheral blood mononuclear cell samples were obtained from patients with metastatic melanoma undergoing monotherapy with ipilimumab via the Interdisciplinary Melanoma Cooperative Group at New York University Langone Medical Center. We isolated CD4+ T-cells and used a cytometric bead array assay following in vitro activation with anti-CD3, anti-CD28 antibodies to characterize cytokine expression profiles by quantifying IFN gamma, IL-2, IL-4, IL-6, IL-10, IL-17, and TNF-α at 5 time points during therapy. In total, 53 peripheral blood samples were included from 12 patients. To correlate cytokine profiles with CD4+ T-cell phenotypes in the intratumoral lymphocyte compartment, multiplex immunofluorescence was performed using CD4, CD8, CCR7, CD45RO, and FOXP3 antibodies on tumors before and after treatment with ipilimumab. RESULTS/ANTICIPATED RESULTS: Patients with evidence of clinical benefit (CB), as defined by having achieved partial response or stable disease, were compared with nonresponders (NR). All patients had an increase in IFN-γ, IL-2, and IL-10 secretion by CD4+ T-cells during ipilimumab therapy. NR had a statistically higher increase in all 3 cytokines. Mean IL-10 secretion was 22.3-fold higher compared with patients with CB (*p* value 0.0458; 95% CI=0.6676–43.89). Mean IFN-γ secretion was 12.4-fold higher from baseline levels in NR compared with CB (*p* value 0.046; 95% CI=0.3589–24.35). Mean IL-2 secretion was 6.9-fold higher in NR compared with CB (*p* value 0.032; 95% CI=0.9688–12.75). There were no statistically significant differences seen in the secretion of IL-4, IL-6, IL-17, or TNF-α. Multiplex immunofluorescence for immune profiling of 20 pre and post treatment tumor biopsies is ongoing. We expect to see distinct intratumoral lymphocyte compartment changes which correlate with clinical response and the above described differential cytokine profiles. Specifically, we anticipate CB patients will have increased intratumoral effector T-cells and decreased regulatory T-cells when compared with their NR counterparts. DISCUSSION/SIGNIFICANCE OF IMPACT: Cytokine expression profiles of peripheral blood CD4+ T-cells have not been previously correlated with patient response in patients undergoing treatment with ipilimumab. We describe distinct secretion profiles for IFN-γ, IL-2, and IL-10 for CB Versus NR patients. NR had a statistically higher increase in IL-10, an inhibitory cytokine which typically indicates upregulation of regulatory T-cells and consequent immune escape. Increased secretion of IL-2 and IFN-γ suggests skewing towards a Th1 type, anti-tumor effector T-cell response; these cytokines increased with ipilimumab treatment in both patient groups. However, the mean increase was several fold higher in NR. Recent evidence suggests loss of the interferon gamma pathway in tumor cells confers resistance to anti-CTLA4 therapy. Chronic IFN-γ secretion is associated with an exhausted T-cell phenotype and impaired tumor rejection. Therefore, higher increases in IFN-γ secretion by CD4+ T-cells in NR suggest impaired IFN-γ dependent tumor rejection in these patients. Our findings suggest IFN-γ, IL-2, and IL-10 cytokine expression profiles can be useful as biomarkers for response to ipilimumab treatment.

